# Preparation and Characterization of a Photo-Crosslinked Methacryloyl-Collagen Composite Film to Promote Corneal Nerve Regeneration via Surface Grafting of Taurine Molecules

**DOI:** 10.3390/ijms241411248

**Published:** 2023-07-08

**Authors:** Yang Liu, Chuanlei Zhang, Yanhui Kong, Huiyu Liu, Cheng Chen, Wenyu Gao, Xiaowei Xi, Hui Yang, Linhong Deng

**Affiliations:** 1 Institute of Biomedical Engineering and Health Sciences, Changzhou University, Changzhou 213164, China; 2 School of Pharmacy, School of Biology and Food Engineering, Changzhou University, Changzhou 213164, China; 3 School of Medical and Health Engineering, Changzhou University, Changzhou 213164, China; 4 School of Medical Information Engineering, Gannan Medical University, Ganzhou 341000, China

**Keywords:** collagen, corneal repair, nerve regeneration

## Abstract

Blindness is frequently caused by corneal abnormalities, and corneal transplantation is the most effective treatment method. It is extremely important to develop high-quality artificial corneas because there are not enough donor corneas accessible for cornea transplantation. One of the most-often utilized materials is collagen, which is the primary component of natural cornea. Collagen-based corneal repair materials have good physicochemical properties and excellent biocompatibility, but how to promote the regeneration of the corneal nerve after keratoplasty is still a big challenge. In this research, in order to promote the growth of nerve cells on a collagen (Col) substrate, a novel collagen-based material was synthesized starting from the functionalization of collagen with unsaturated methacryloyl groups that three-dimensionally photopolymerize to a 3D network of chemically crosslinked collagen (ColMA), onto which taurine molecules were eventually grafted (ColMA-Tr). The physicochemical properties and biocompatibility of the Col, ColMA and ColMA-Tr films were evaluated. By analyzing the results, we found that all the three samples had good moisture retention and aq high covalent attachment of methacryloyl groups followed by their photopolymerization improved the mechanical properties of the ColMA and ColMA-Tr. Most importantly, compared with ColMA, the taurine-modified collagen-MA film significantly promoted the growth of nerve cells and corneal epithelial cells on its surface. Our preliminary results suggest that this novel ColMA-Tr film may have potential use in cornea tissue engineering in the future.

## 1. Introduction

The cornea plays a crucial function in preserving the health of the eyeball and maintaining good eyesight [1]. It is located in the anterior portion of the eyeball. Globally, corneal disease is a common cause of blindness, and keratoplasty is seen to be the most successful treatment to regain vision [2]. However, for keratoplasty, the major problem is the severe shortage of donor corneas and high-quality artificial corneas [3]. To-date, various corneal repair materials such as decellularized cornea [4], acrylate artificial cornea [1], amniotic membranes [5], and natural polymer-based materials (gelatin [6], chitosan [7], collagen [8], etc.) have been developed and utilized to treat corneal blindness. Among those natural polymers, collagen has been extensively studied as the preferred material for corneal repair, because it possesses suitable physicochemical properties and biological characteristics similar to the native cornea [9]. At present, several collagen-based material has been reported to have achieve some specific functions, such as good biocompatibility [10], antibacterial effect (when adequate antimicrobial is incorporated) [11] and moisturizing performance [12]. But how to promote the regeneration of corneal nerve tissue in collagen-based artificial cornea after keratoplasty is still a big challenge.

As is well known, corneal nerves play a critical role in controlling corneal development and preserving the homeostasis of the ocular surface. Reduced tear production, impaired epithelial function, and even corneal hypoesthesia are all results of abnormal corneal innervation [13,14]. For collagen-based cornea repair materials, it is of great significance to promote the regeneration of the corneal nerve for the recovery of corneal function. Taurine (Tr), an important neuro-repair molecule in the visual system [15], is involved in several significant physiological functions, such as incorporating antioxidant activity, the maintenance of cell morphology, anti-inflammatory activity, and so on [16,17]. Currently, the frequent dosing of functional drugs or growth factors that are commonly used to promote nerve repair is a normal way to treat eyes after corneal transplantation, but the bio-availability of the drugs is poor, and the dosage of drugs is difficult to control [18]. Furthermore, the optical properties of corneal repair materials embedded with drugs by physical methods are decreased due to the structural changes of scaffolds [19]. Crucially, it is worth mentioning that the functional groups of the taurine molecule provide a new method for chemical grafting to the surface of the collagen-based film [20]. Therefore, in this study, we attempted to introduce taurine molecules on the surface of collagen-based film through chemical modification.

On the other hand, excellent mechanical properties are vital for a successful corneal implant [21]. Although collagen (Col) is highly biocompatible [22], the low mechanical properties of collagen-based film often lead to material intolerance to tear and destruction, which limits its application [23]. In order to improve the mechanical properties of collagen film, silk fibroin [24], poly-rotaxane multiple aldehyde [25], cellulose nanocrystals [26] and other components were introduced into collagen-based materials. Methacrylic anhydride (MA), a biosafe photo-crosslinking molecule, has been reported to improve the mechanical properties of many biomaterials [27], including gelatin [28], chitosan [29], poly(epsilon-caprolactone) [30], carboxymethyl chitin [31], etc. For example, a chemically modified MA-gelatin with abundant double-bonded groups, can form a stable network structure under ultraviolet irradiation, which is attractive for us to enhance the collagen-based materials [32].

Therefore, in order to address the mechanical weakness, we first utilized an MA molecule to chemically crosslink with a collagen molecule. The collagen-based film’s surface was then chemically grafted with taurine molecules. After that, the study assessed the physicochemical characteristics and biocompatibility of the Col, ColMA, and ColMA-Tr. The effect of the ColMA-Tr on encouraging nerve healing and corneal epithelialization was further examined using PC12 nerve cells and human corneal epithelial cells (HCECs).

## 2. Results and Discussion

### 2.1. Infrared Spectroscopy Analysis

**Figure 1:**
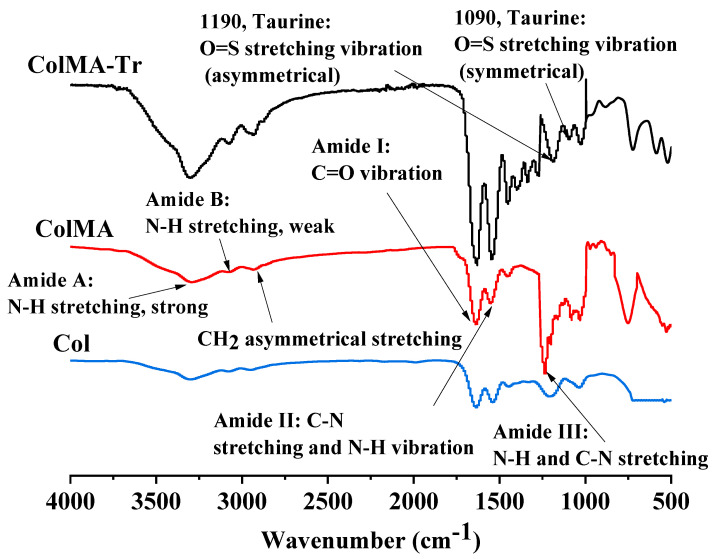
The FTIR spectra of the Col, ColMA, and ColMA-Tr films in the wavenumber range of 4000-500 cm^−1^. ColMA-Tr film showed characteristic absorption peaks of taurine molecules at 1190 cm^−1^ and 1090 cm^−1^, which confirmed that taurine was successfully grafted onto the surface of ColMA-Tr film.

The FTIR spectra of the three films are shown in Figure 1. They reveal a number of spectral features characteristic to the collagen structure: amide A (N-H stretching, strong) and amide B (N-H stretching, weak) bands located around 3310 cm^−1^ and 3070 cm^−1^, respectively; asymmetric stretching vibrations of C-H (mostly belonging to methylene groups and, to a lesser extent, to methyl groups) at about 2930 cm^−1^; amide I (C=O stretching), II (a mixture of C-N stretching and N-H bending), and III (C-N stretching, N-H bending) absorption bands at 1630 cm^−1^, 1550 cm^−1^ and 1220 cm^−1^, respectively [33,34]. Inserting methacryloyl entities onto collagen molecules via amide linkages and then completing the three-dimensional polymerization of these unsaturated structures enhanced the relative intensity of the absorption bands regarding C=O and C-H (-CH2- and -CH3 groups) stretching vibrations as can be seen on the IR spectrum of ColMA. Instead, the FTIR spectrum of ColMA-Tr displays two more absorption bands at 1190 cm^−1^ and 1090 cm^−1^ ascribed to the asymmetric and symmetric stretching of S=O bonds of -SO_3_- groups [35] at physiological pH, which is strong evidence of the successful synthesis of the final collagen-based hydrogel functionalized with taurine (ColMA-Tr).

### 2.2. Evaluation of Contact Angle and Water Absorption

**Figure 2:**
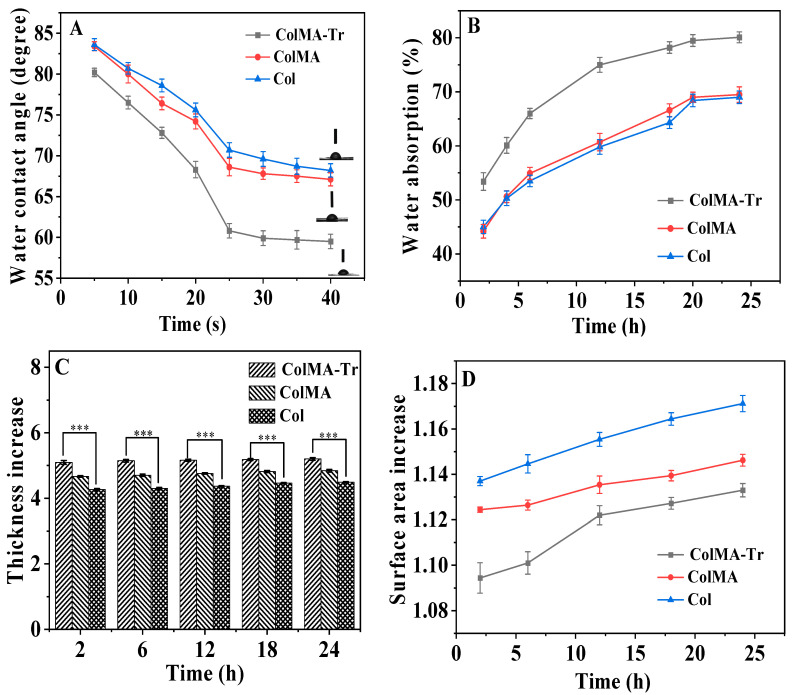
Water absorption and moisture-retention properties of Col, ColMA, ColMA-Tr films: variation of the water contact angle (**A**), water absorption (**B**), thickness (**C**), and surface size (**D**) versus time. The original thickness of the films was about 22.4 ± 1.1 μm, and the original surface size of the films was about 1.3 ± 0.2 cm^2^ (*n* = 5). *** *p* < 0.01, which were considered to be statistically significant.

Good water absorption and moisture are known to preserve the wettability of the eye’s surface and lower the risk of xerophthalmia, making them essential characteristics of the cornea [21]. The wettability of films is assessed by contact angle measurements (Figure 2A). The contact angle values of the ColMA-Tr film fell within 80.2 ± 0.5° to 59.5 ± 0.9° interval, indicating the increased hydrophilicity of this surface. We detected considerably lower contact angle values for the ColMA-Tr film compared to Col as well as ColMA film. Within 24 h, all collagen-based films’ water absorption basically attained a stable state of saturation (Figure 2B). Collagen-based material’s hydrophilicity was not substantially influenced by crosslinking induced by three-dimensional polymerization of methacryloyl groups grafted onto the collagen structure. However, the wettability of the film was improved through taurine grafting. The water absorption of ColMA-Tr film is 80.1 ± 1.0%, which is quite similar to that of human cornea [36]. Notably, a qualified artificial cornea should be reproducible and controllable and prepare the appropriate size easily, which can provide more options for corneal blind patients [37]. ColMA-Tr film expanded in thickness by 520% when the materials reached the saturated water-absorption condition, a significant higher value compared with those obtained for Col (449%) and ColMA (484%) films (Figure 2C). Additionally, there was little variation in the surface size of the three films (Figure 2D). The aforementioned findings demonstrate that ColMA-Tr films have a high degree of hydrophilicity and moisture retention, which may be related to the taurine moieties grafted onto the collagen structure of the crosslinked ColMA films, which means a large number of sulfonic/sulfonate groups and the same number of amide-crosslinkages resulting after carbodiimide-mediated coupling/grafting reaction [38]. Instead, collagen functionalization with MA followed by crosslinking polymerization had no discernible impact on the hydrophilicity of collagen-based films. In addition, the films display a dense structure and their size can be controlled in both the dry and wet state, making these systems easily adaptable to various morphologies.

### 2.3. Optical Performance Evaluation

**Figure 3:**
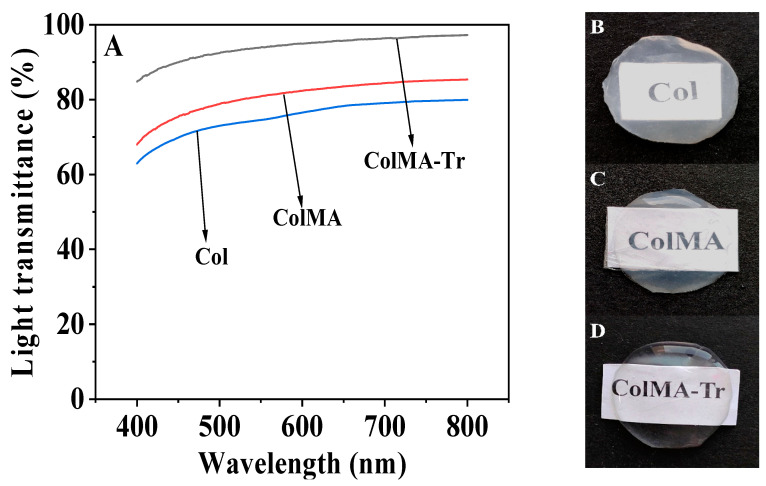
Optical properties of Col, ColMA, ColMA-Tr film after saturated water absorption: transmittance curve over the wavelength range of 400–800 nm (**A**), and images of Col (**B**), ColMA (**C**), ColMA-Tr (**D**) films investigated.

Good optical properties (high light transmittance, among others) are one of the key features for a physiologically functioning corneal tissue [39]. The films we fabricated in this study showed excellent transparency and matched the cornea’s optical property in its physiological condition (Figure 3). All of the three samples’ transmittances rose as the wavelength increased. In the 300–800 wavelength range, the ColMA-Tr film demonstrated a light transmittance of over 90%, while in comparison, the Col film had a substantially lower value. Digital images also demonstrate that ColMA and ColMA-Tr films have high transparency than Col film. Because of its uniform structure and high light transmittance, ColMA-Tr film has a promising potential of being used for corneal repair.

### 2.4. Mechanical Property Analysis

**Figure 4:**
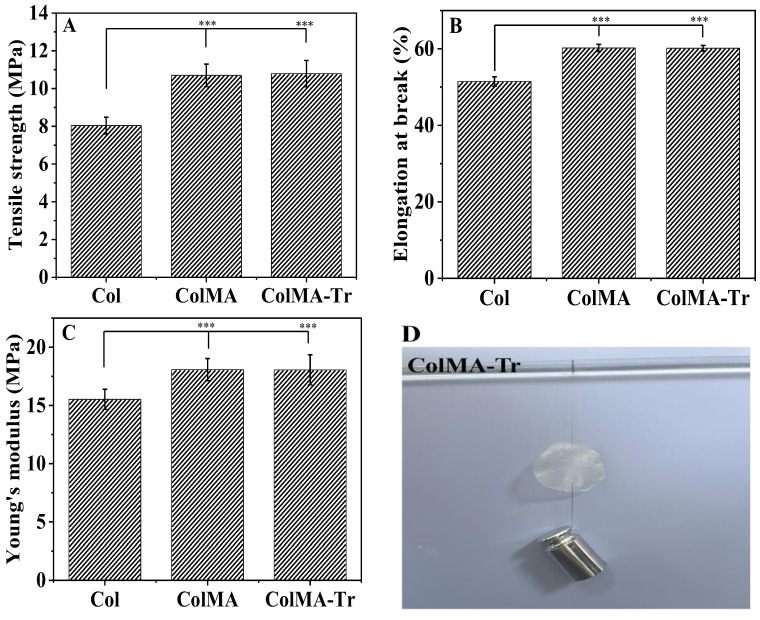
Mechanical properties of Col, ColMA and ColMA-Tr films: tensile strength (**A**), elongation at break (**B**) and Young’s modulus (**C**); the ColMA-Tr film is suture-resistant to a 10-0 ophthalmic suture (**D**) (*n* = 5). Compared with Col film, the mechanical properties of ColMA film and ColMA-Tr films are significantly improved, and also exceed those of natural human corneal tissue. *** *p* < 0.01, which were considered to be statistically significant.

Maintaining the normal structure of the eyeball and intraocular osmotic pressure depends heavily on the maintenance of good mechanical properties of the cornea [40]. Good suture resistance of the material during keratoplasty can significantly increase the success probability of the surgical procedure [32]. Col film was utilized as the blank control group in the wet tensile tests (Figure 4A–C). Both ColMA (10.70 ± 0.60 MPa) and ColMA-Tr (10.79 ± 0.70 MPa) had almost the same values of tensile strength, but they were significantly higher than that of the blank Col group (8.04 ± 0.44 MPa). The same tendency was noticed for the quantities of Young’s modulus and the elongation at break. According to our calculations, the ColMA-Tr film’s Young’s modulus was 18.06 ± 1.29 MPa, a value higher than that of the Col films by ca. 3 MPa. Practically, these mechanical properties of the ColMA-Tr were found to be better than those of healthy human corneal tissue. The ColMA-Tr film can tolerate 10-0 surgical sutures, according to in vitro suture experiments (Figure 4D). Based on these results, the grafting of taurine on the collagen-based film surface exhibited minimal impact on the mechanical properties of the samples, but inserting the methacryloyl moieties greatly improved both the tensile strength and Young’s modulus of the final system ColMA after three-dimensional polymerization. In other words, the unsaturated methacryloyl groups chemically anchored onto collagen molecules were able to polymerize (via radical polymerization under UV irradiation), leading to more, shorter crosslinkages between collagen entities randomly spread throughout the volume of the system that finally became a chemically crosslinked hydrogel.

### 2.5. Analysis of Drug Loading and Sustained Release of ColMA-Tr Film

**Figure 5:**
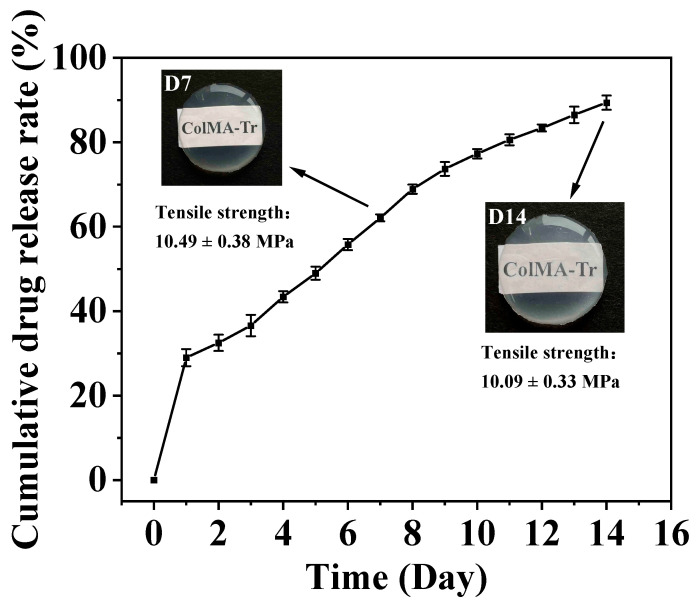
Cumulative drug release of ColMA-Tr film. The taurine release from the ColMA-Tr film lasts for more than two weeks (*n* = 5).

The detection of drug-release and drug-load behavior is critical for predicting in vivo performance for quality control [41]. Hence, the drug loading of ColMA-Tr film and the cumulative drug release at different time points (Figure 5) were detected. The taurine load of ColMA-Tr film was calculated to be 2.42 ± 0.28%. The ColMA-Tr film displayed a long-term release pattern for two weeks, and in the first day of release, roughly 30% of the taurine contained in the film was released, providing an appropriate drug dosage for the beginning of treatment. The subsequent stage saw a slower, continuous medication release rate. By day 14, the total amount of taurine released by the film exceeded 89%. As a result, we think that ColMA-Tr film has a good drug-release function, can maintain stable drug concentration, has a high bioavailability, and is helpful in lowering medication toxicity and side effects. In addition, the mechanical and optical properties of ColMA-Tr film did not alter significantly after 14 days of sustained taurine release (Figure 5), which may be due to the fact that taurine molecules were mainly grafted on the surface of the ColMA-Tr film, and the drug release process did not considerably damage the physical structure of this collagen-based material.

### 2.6. Growth of PC12 Nerve Cells on ColMA and ColMA-Tr Film

**Figure 6:**
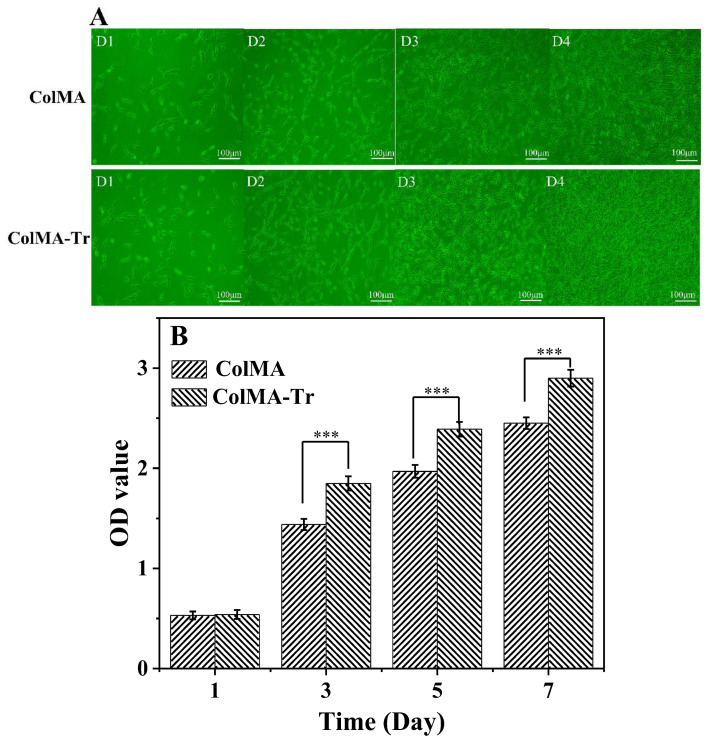
Morphological changes of PC12 cells on the surface of ColMA and ColMA-Tr at 1, 2, 3, and 4 days (**A**). MTT analysis of cell proliferation on ColMA and ColMA-Tr films at 1, 3, 5 and 7 days after seeding (*n* = 5, measured at 490 nm) (**B**). Both of ColMA and ColMA-Tr films displayed a good cytocompatibility, but ColMA-Tr film significantly promoted the growth of PC12 cells, most likely due to the presence of grafted taurine entities. *** *p* < 0.01, which were considered to be statistically significant.

The growth and proliferation of PC12 nerve cells has been employed in numerous investigations to assess the ability of materials to support neural repair [42]. To verify our hypothesis, we tested the cytocompatibility of ColMA and ColMA-Tr film on different days. The morphology of the PC12 cells changed from round to long fusiform after they have adhered to the collagen-based films well (Figure 6A). After 4 days of incubation, ColMA-Tr film was almost entirely covered with PC12 cells, and the cell density was much higher than that of ColMA film. Using the MTT assay, the proliferation and cytotoxicity of PC12 cells on films were assessed (Figure 6B). Compared with ColMA film, cell growth was quicker and better when cultivated on ColMA-Tr film, and the optical density (OD) of the cell suspension rose with the incubation time. These findings show that the innovative ColMA-Tr film is more suitable for PC12-cell growth and proliferation. We believe that the addition of taurine molecules enhanced not only the growth and multiplication of PC12 cells but also the deposition of the extracellular matrix, further creating an adequate milieu for cell development.

### 2.7. Adhesion and Proliferation of HCECs on ColMA and ColMA-Tr Film

**Figure 7:**
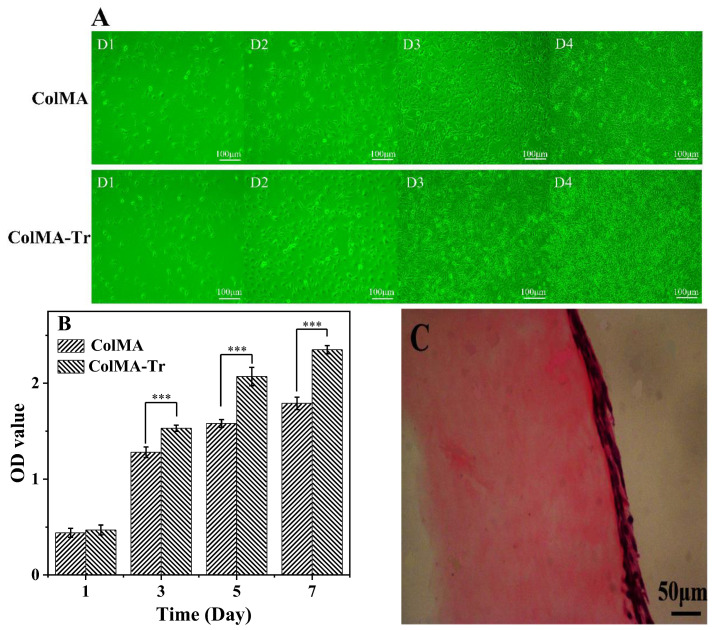
Growth and proliferation of HCECs on the surface of ColMA and ColMA-Tr: cell adhesion and growth at 1, 2, 3, and 4 days (**A**), MTT assay results of HCEC proliferation on collagen-based films after incubation at days 1, 3, 5 and 7 (*n* = 5) (**B**). *** *p* < 0.01, which were considered to be statistically significant. After 7 days, the H and E staining of the ColMA-Tr film section showed that the surface of the film is covered with 2–3 layers of HCECs (**C**).

The control of the human eye’s homeostasis depends on the corneal epithelium, and corneal epithelial cells are significant components of preclinical models used to evaluate ophthalmic products or medications [43]. The excellent biocompatibility of Col film has already been demonstrated [38]. In order to evaluate the biocompatibility of the ColMA and ColMA-Tr film with better mechanical properties, HCECs were used in this investigation. On the surface of the two collagen-based materials, we found that HCECs had a favorable growth state, and the cells gradually changed from their initial spherical shape to the natural normal shape. The growth rate of the HCECs was higher on the ColMA-Tr surface than that noticed on the ColMA surface (Figure 7A). After seven days of cultivation, the MTT assay results revealed that the two films had no harmful effects on HCECs (Figure 7B). From day 3 to day 5, there was a noticeable increase in the number of HCECs, and this growth persisted until day 7. The outcomes showed that the presence of grafted taurine functionalities onto the ColMA surface (ColMA-Tr) could favor the human corneal epithelial cells to adhere and proliferate on collagen-based films. The surface of the ColMA-Tr film was found to have continuous 2–3 layers of cells, similar to the natural superior cortex on the human cornea, according to histological inspection of cross-sectional H and E staining sections (Figure 7C). According to the experimental findings, we believe that the ColMA-Tr possesses a high cytocompatibility, which opens up more options for its use in corneal repair when combined with the aforementioned investigations.

## 3. Materials and Methods

### 3.1. Materials

Type I collagen was extracted from bovine Achilles tendon. Taurine and paraformaldehyde were bought from the American Sigma Chemical Company. The MA was provided by Aladdin Biochemical Technology Co., Ltd. (Shanghai, China). Suzhou Intelligent Manufacturing Research Institute (Suzhou, China) provided the photo-initiator LAP. Maclean Biochemical Technology Co., Ltd. (Shanghai, China) provided the Ethyl-3-(3-dimethyl aminopropyl) carbodiimide (EDC) and N-hydroxysuccinimide (NHS). PBS buffer (pH = 7.4) was prepared from powder reagent form (Shanghai, China). Thermo Fisher Technology Co., Ltd. (Shanghai, China) provided all of the cell culture-related reagents used in this investigation. The PC12 neurons utilized in this study were provided by the Chinese Academy of Sciences culture collection in Shanghai, China. The School of Optometry and the Eye Hospital of Wenzhou Medical University in China provided HCECs for this study. All other chemicals in this study were of analytical grade.

### 3.2. Preparation Method of ColMA-Tr Film

**Figure 8:**
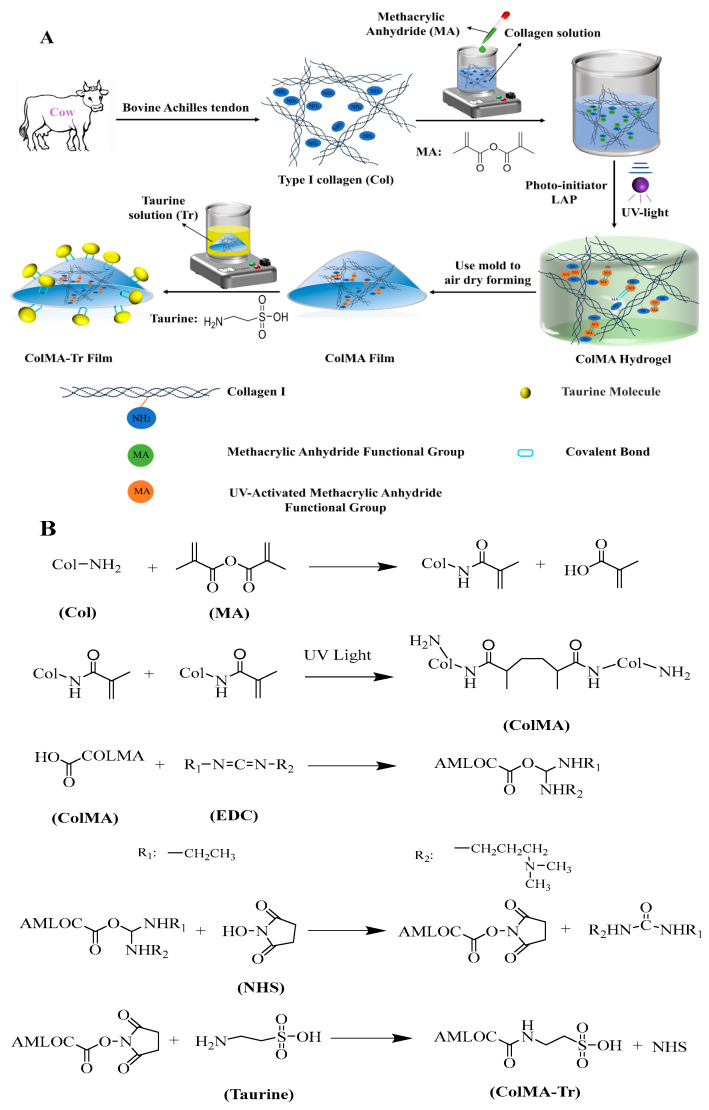
Schematic diagram of the synthesis of ColMA-Tr film (**A**), and its chemical crosslinking mechanism (**B**).

ColMA gel was fabricated using the previously disclosed GelMA preparation [28]. Briefly, the methacrylic anhydride was added to the collagen solution drop by drop at a ratio of collagen: methacrylic anhydride = 1 g: 0.1 mL for overnight reaction before being dialyzed. In the following 20 min, 20% (*v/v*) LAP was added to the sterilized ColMA solution. To create a cornea-shaped ColMA film, the composite solution was then air-dried in a specific mould under sterile circumstances after being exposed to UV (wavelength of 365 nm) radiation. The produced ColMA film was then washed with distilled water three times, dried in a sterile environment, and submerged in an aqueous solution of taurine (mass ratio of EDC: NHS: taurine is 1:1:6) for a four-hour crosslinking reaction. Figure 8 displays schematically the synthesis route of the ColMA-Tr film.

### 3.3. Fourier Transform Infrared Spectroscopy

By using a Fourier Transform Infrared (FTIR) spectrometer equipped with a module of Attenuated Total Reflectance (ATR) (FTIR-ATR instrument Vector 33, Bruker, Germany), the IR spectra of Col, ColMA and ColMA-TR in the wavenumber range of 4000 to 500 cm^−1^ were acquired.

### 3.4. Contact Angle and Swelling Property Tests

Utilizing a JC2000D1 contact angle measuring apparatus (Zhongchen Digital Co., Ltd, Shanghai, China), the contact angle of Col, ColMA, and ColMA-Tr film was studied. A volume of 2 µL water was dripped onto the surface of the three samples and then the values of static contact angle were assessed at predefined time intervals. Each individual value was obtained as a mean of 5 trials.

The water absorption of Col, ColMA and ColMA-Tr film was measured by swelling them in PBS (pH = 7.4) at 37 °C. After using filter paper to gently wipe the film surface, the samples were immediately weighed to determine their wet weight. Samples of defined diameters were swollen in PBS, and at predetermined time intervals, the thickness and surface area of the hydrated films were measured. The thickness of the sample was determined by a spiral micrometer, while the surface area of the film was obtained by calculating the surface area of the square sample. Each final numerical result was the average of five trials. The water absorption, thickness and surface size of the films were calculated according to Relationships (1)–(3):

(1) $$\mathrm{Water}\;\mathrm{absorption}\;(\%)=((W_{t}-W_{0})/W_{t})\times 100$$

(2) $$\mathrm{Thickness}\;\mathrm{increase}=H_{t}/H_{0}$$

(3) $$\mathrm{Surface}\;\mathrm{area}\;\mathrm{increase}=S_{t}/S_{0}$$

where W_0_, H_0_ and S_0_ indicate the initial weight and size of the films, respectively, while W_t_, H_t_ and S_t_ represent the final weight and sizes of the wet samples, respectively.

### 3.5. Light Transmittance

Each pre-wet sample’s light transmittance was evaluated using a UV-2450 UV-visible spectrophotometer (Shimadzu Co., Ltd., Shanghai, China). The transmittance of light through the films was measured at wavelengths ranging from 400 to 800 nm and a constant wavelength step of 1 nm. Prior to measurement, the films were hydrated in PBS and the transmittance of the films was adjusted with a blank consisting of distilled water.

### 3.6. Mechanical Properties Measurement

The Col, ColMA, and ColMA-Tr films were submerged in PBS for 24 h before performing tensile experiments by employing the Bose ELF3200 mechanical testing equipment (USA). Each result of the study was the average of 5 trials. Additionally, ColMA-Tr film was subjected to in vitro suture testing using surgical sutures, which were imagistically assessed.

### 3.7. Assessment of Drug Loading and In Vitro Drug Release

The ColMA-Tr film’s taurine release was tested in PBS with a pH of 7.4 and a constant temperature of 37 °C. The 2 mL of drug release solution was removed and put away at various intervals, and 2 mL of new PBS supplement solution was added. After that, a UV-2800A UV-visible spectrophotometer (China) was used to examine the amount of taurine present in the release solution [44]. Five parallel experiments were run by each group. The drug loading of ColMA-Tr film was calculated according to Formulae (4) and (5), and the cumulative drug release was shown in Formula (6):

(4) $$A_{1}/C_{a}=A_{2}/C_{b}$$

(5) $$\mathrm{Taurine}\;\mathrm{grafting}\;\mathrm{rate}=(C_{a}-C_{b})V/M$$

(6) $$\mathrm{Cumulative}\;\mathrm{release}\;\mathrm{fraction}\;\mathrm{of}\;\mathrm{taurine}\;(\%)=((V_{e}\;\sum_{1}^{n-1}C_{i}\;+V_{0}C_{n})/W_{0})\times 100$$

Here, A_1_ and C_a_ are, respectively, the absorbance value and concentration of taurine solution before reaction, A_2_ and C_b_ are, respectively, the absorbance value and concentration of taurine solution after reaction, V is the volume of taurine solution, and M is the mass of ColMA film. In addition, V_e_ is the amount of PBS taken out each time, V_0_ is the total volume of PBS, C_i_ and C_n_ are the content of taurine at different time points, W_0_ is the load of taurine on ColMA-Tr film.

### 3.8. Adhesion and Proliferation of PC12 Cells on ColMA and ColMA-Tr Films

PC12 cells were incubated in DMEM (Gibco BRL; Dulbecco’s Modified Eagle’s Medium), which also contained 10% fetal bovine serum (FBS), 100 g/mL streptomycin, and 100 units/mL penicillin. PC12 cells were cultivated in a 37 °C incubator with 5% CO_2_ atmosphere. The ColMA and ColMA-Tr film were placed in a 6-well plate and disinfected before the cell experiments. PC12 cells with a normal physiological state were inoculated on the surface of the films and incubated with complete culture medium, and the cell morphology was monitored after incubation for 1 d, 2 d, 3 d and 4 d. Moreover, MTT assay was used to detect the proliferative activity of PC12 cells.

### 3.9. HCECs Growth on the Surface of ColMA and ColMA-Tr Film

ColMA-Tr films were placed in a 6-well plate containing sterilized ColMA, and the human corneal epithelial cell suspensions in a good growth condition were poured in. Then, at 37 °C and 5% CO_2_, HCECs were cultured in complete media that contained DMEM supplemented with 10% FBS, 5 g/mL insulin, 100 g/mL streptomycin and 100 units/mL penicillin. Every two days, the culture media was replaced. At the same time, a fluorescent microscope (EVOS FL, Thermo Fisher Scientific) was used to observe the morphological change in the cells on the samples every day. MTT assay was additionally carried out to evaluate the proliferation of HCECs.

### 3.10. Histology

After seven days of HCEC culture on the surface of ColMA-Tr films, the samples were then fixed with 4% paraformaldehyde at room temperature for 36 h, dehydrated at a gradient with ethanol and xylene, and finally embedded in paraffin wax. Slices of 10 micrometers thick were prepared with standard hematoxylin and eosin (H&E) staining.

### 3.11. Statistical Analysis

Data are expressed as mean ± standard deviation. A T test was used for statistical analysis to determine the degree of significance. ** *p* < 0.05 and *** *p* < 0.01 were considered to be statistically significant.

## 4. Conclusions

The findings suggest that the three-dimensional polymerization of the grafted methacryloyl groups onto the collagen triple helices substantially enhances the collagen film’s mechanical properties, and the results according to cell-culture experiments demonstrate that the anchoring of taurine functionalities onto the crosslinked ColMA structure significantly promoted the growth of nerve cells and corneal epithelization. In addition, this novel functional collagen-based film intended for corneal reconstruction was found to be very swellable and hydrophilic in an aqueous environment, which lowers the risk of xerophthalmia after transplantation. Moreover, this taurine-functionalized collagen-based film in its crosslinked condition revealed excellent optical properties comparable to human corneas. Thus, we conclude that the newly obtained ColMA-Tr material, which is simple to manufacture and non-toxic, could have a huge potential use for corneal repair in the future.

## Data Availability

Data are included in the text; raw data are available from the corre-sponding authors.

## References

[1] Palchesko R.N., Carrasquilla S.D., Feinberg A.W. (2018). Natural Biomaterials for Corneal Tissue Engineering, Repair, and Regeneration. Adv. Healthc. Mater..

[2] Liu C., Saeed H.N. (2023). Disparities in Access to Corneal Tissue in the Developing World. Semin. Ophthalmol..

[3] Holland G., Pandit A., Sánchez-Abella L., Haiek A., Loinaz I., Dupin D., Gonzalez M., Larra E., Bidaguren A., Lagali N. (2021). Artificial Cornea: Past, Current, and Future Directions. Front. Med..

[4] Du L., Wu X., Pang K., Yang Y. (2011). Histological Evaluation and Biomechanical Characterisation of an Acellular Porcine Cornea Scaffold. Br. J. Ophthalmol..

[5] Kumar P., Pandit A., Zeugolis D.I. (2016). Progress in Corneal Stromal Repair: From Tissue Grafts and Biomaterials to Modular Supramolecular Tissue-Like Assemblies. Adv. Mater..

[6] Goodarzi H., Jadidi K., Pourmotabed S., Sharifi E., Aghamollaei H. (2019). Preparation and in Vitro Characterization of Cross-Linked Collagen–Gelatin Hydrogel Using EDC/NHS for Corneal Tissue Engineering Applications. Int. J. Biol. Macromol..

[7] Dong Q., Wu D., Li M., Dong W. (2022). Polysaccharides, as Biological Macromolecule-Based Scaffolding Biomaterials in Cornea Tissue Engineering: A Review. Tissue Cell.

[8] Kong B., Sun L., Liu R., Chen Y., Shang Y., Tan H., Zhao Y., Sun L. (2022). Recombinant Human Collagen Hydrogels with Hierarchically Ordered Microstructures for Corneal Stroma Regeneration. Chem. Eng. J..

[9] Chen Y., Sun X., Peng Y., Eichenbaum J.V., Ren L., Liu Y. (2022). Effects of Different Radiation Sources on the Performance of Collagen-Based Corneal Repair Materials and Macrophage Polarization. ACS Omega.

[10] Jung O., Radenkovic M., Stojanović S., Lindner C., Batinic M., Görke O., Pissarek J., Pröhl A., Najman S., Barbeck M. (2020). In Vitro and In Vivo Biocompatibility Analysis of a New Transparent Collagen-Based Wound Membrane for Tissue Regeneration in Different Clinical Indications. In Vivo.

[11] Liu Y., Ren L., Long K., Wang L., Wang Y. (2014). Preparation and Characterization of a Novel Tobramycin-Containing Antibacterial Collagen Film for Corneal Tissue Engineering. Acta Biomater..

[12] Liu Y., Lv H., Ren L., Xue G., Wang Y. (2016). Improving the Moisturizing Properties of Collagen Film by Surface Grafting of Chondroitin Sulfate for Corneal Tissue Engineering. J. Biomater. Sci. Polym. Ed..

[13] Liu C.Y., Arteaga A.C., Fung S.E., Cortina M.S., Leyngold I.M., Aakalu V.K. (2021). Corneal Neurotization for Neurotrophic Keratopathy: Review of Surgical Techniques and Outcomes. Ocul. Surf..

[14] Cruzat A., Qazi Y., Hamrah P. (2017). In Vivo Confocal Microscopy of Corneal Nerves in Health and Disease. Ocul. Surf..

[15] Militante J.D., Lombardini J.B. (2002). Taurine: Evidence of Physiological Function in the Retina. Nutr. Neurosci..

[16] Taurine Alleviates Chronic Social Defeat Stress-Induced Depression by Protecting Cortical Neurons from Dendritic Spine Loss|SpringerLink. https://link.springer.com/article/10.1007/s10571-022-01218-3.

[17] Ramírez-Guerrero S., Guardo-Maya S., Medina-Rincón G.J., Orrego-González E.E., Cabezas-Pérez R., González-Reyes R.E. (2022). Taurine and Astrocytes: A Homeostatic and Neuroprotective Relationship. Front. Mol. Neurosci..

[18] Li X., Nie S., Kong J., Li N., Ju C., Pan W. (2008). A Controlled-Release Ocular Delivery System for Ibuprofen Based on Nanostructured Lipid Carriers. Int. J. Pharm..

[19] Ran W., Ma H., Li M. (2020). In Vitro and In Vivo Studies of Polyvinyl Pyrrolidone–Coated Sparfloxacin-Loaded Ring Contact Lens to Treat Conjunctivitis. J. Pharm. Sci..

[20] Yao Q.Y., Wang L., Du Z., Li K., Lin Y. (2013). Characterization and Biological Evaluation of Structurally Modified Taurine Using Benzaldehydes. Asian J. Chem..

[21] Luo Y., Li G., Chen L., Hong F.F. (2023). Preparation and Evaluation of Bacterial Nanocellulose/Hyaluronic Acid Composite Artificial Cornea for Application of Corneal Transplantation. Biomacromolecules.

[22] Li H.C., Sun X.M., Huang Y.R., Peng Y.H., Liu J., Ren L. (2022). Synthetic Crosslinker Based on Amino–Yne Click to Enhance the Suture Tension of Collagen-Based Corneal Repair Materials. ACS Appl. Polym. Mater..

[23] Dong L., Liu Q., Gao Y., Jia H., Dai W., Guo L., Fan H., Fan Y., Zhang X. (2021). The Effect of Collagen Hydrogels on Chondrocyte Behaviors through Restricting the Contraction of Cell/Hydrogel Constructs. Regen. Biomater..

[24] Long K., Liu Y., Li W., Wang L., Liu S., Wang Y., Wang Z., Ren L. (2015). Improving the Mechanical Properties of Collagen-Based Membranes Using Silk Fibroin for Corneal Tissue Engineering: Improving the Mechanical Properties of Collagen-Based Membranes. J. Biomed. Mater. Res. A.

[25] Lei X., Jia Y.G., Song W., Qi D., Jin J., Liu J., Ren L. (2019). Mechanical and Optical Properties of Reinforced Collagen Membranes for Corneal Regeneration through Polyrotaxane Cross-Linking. ACS Appl. Bio Mater..

[26] Qin L., Gao H., Xiong S., Jia Y., Ren L. (2020). Preparation of Collagen/Cellulose Nanocrystals Composite Films and Their Potential Applications in Corneal Repair. J. Mater. Sci. Mater. Med..

[27] Rosiak J.M., Yoshii F. (1999). Hydrogels and Their Medical Applications. Nucl. Instrum. Methods Phys. Res. Sect. B.

[28] Farasatkia A., Kharaziha M., Ashrafizadeh F., Salehi S. (2021). Transparent Silk/Gelatin Methacrylate (GelMA) Fibrillar Film for Corneal Regeneration. Mater. Sci. Eng. C.

[29] Hu Y., Xu Y., Wang B., Chen Y., Huang C. (2022). Facile Preparation and Characterization of Photopolymerized Adhesive Hydrogels Based on Methacrylated Catechol-Chitosan. J. Mater. Sci..

[30] Elomaa L., Teixeira S., Hakala R., Korhonen H., Grijpma D.W., Seppälä J.V. (2011). Preparation of Poly(ε-Caprolactone)-Based Tissue Engineering Scaffolds by Stereolithography. Acta Biomater..

[31] Kang W., Bi B., Zhuo R., Jiang X. (2017). Photocrosslinked Methacrylated Carboxymethyl Chitin Hydrogels with Tunable Degradation and Mechanical Behavior. Carbohydr. Polym..

[32] Yang X., Sun X., Liu J., Huang Y., Peng Y., Xu Y., Ren L. (2021). Photo-Crosslinked GelMA/Collagen Membrane Loaded with Lysozyme as an Antibacterial Corneal Implant. Int. J. Biol. Macromol..

[33] Coates J., Meyers R.A. (2000). Interpretation of Infrared Spectra, A Practical Approach. Encyclopedia of Analytical Chemistry.

[34] Larkin P. (2011). Infrared and Raman Spectroscopy: Principles and Spectral Interpretation.

[35] Pircher M., Götzinger E., Leitgeb R., Fercher A.F., Hitzenberger C.K. (2003). Measurement and Imaging of Water Concentration in Human Cornea with Differential Absorption Optical Coherence Tomography. Opt. Express.

[36] Dragan E.S. (2014). Advances in Interpenetrating Polymer Network Hydrogels and Their Applications. Pure Appl. Chem..

[37] Liu Y., Zhang C., Kong Y., Liu H., Guo J., Yang H., Deng L. (2022). Modification of Collagen Film via Surface Grafting of Taurine Molecular to Promote Corneal Nerve Repair and Epithelization Process. J. Funct. Biomater..

[38] Lai J.Y., Li Y.T., Cho C.H., Yu T.C. (2012). Nanoscale Modification of Porous Gelatin Scaffolds with Chondroitin Sulfate for Corneal Stromal Tissue Engineering. Int. J. Nanomed..

[39] Kong B., Mi S. (2016). Electrospun Scaffolds for Corneal Tissue Engineering: A Review. Materials.

[40] Knoke S., Bunjes H. (2021). Transfer of Lipophilic Drugs from Nanoemulsions into Lipid-Containing Alginate Microspheres. Pharmaceutics.

[41] Chen T., Jiang H., Zhu Y., Chen X., Zhang D., Li X., Shen F., Xia H., Min Y., Xie K. (2021). Highly Ordered 3D Tissue Engineering Scaffolds as a Versatile Culture Platform for Nerve Cells Growth. Macromol. Biosci..

[42] Zhang J., Zhang C.W., Du L.Q., Wu X.Y. (2016). Acellular Porcine Corneal Matrix as a Carrier Scaffold for Cultivating Human Corneal Epithelial Cells and Fibroblasts In Vitro. Int. J. Ophthalmol..

[43] Tian F., Li S. (2020). Study of Spectrophotometric Characteristics of the Charge Transfer Complex of Taurine Drug with 7,7,8,8-Tetracyanoquinodimethane. Quimica Nova.

[44] Brăzdaru L., Staicu T., Albu Kaya M.G., Chelaru C., Ghica C., Cîrcu V., Leca M., Ghica M.V., Micutz M. (2022). 3D Porous Collagen Matrices—A Reservoir for In Vitro Simultaneous Release of Tannic Acid and Chlorhexidine. Pharmaceutics.

